# Cigar Warning Noticing and Demographic and Usage Correlates: Analysis from the United States Population Assessment of Tobacco and Health Study, Wave 5

**DOI:** 10.3390/ijerph19063221

**Published:** 2022-03-09

**Authors:** Stefanie K. Gratale, Arjun Teotia, Julia Chen-Sankey, Ollie Ganz, Cristine D. Delnevo, Andrew A. Strasser, Olivia A. Wackowski

**Affiliations:** 1 Rutgers Center for Tobacco Studies, Rutgers Biomedical and Health Sciences, Rutgers University, New Brunswick, NJ 08901, USA; at1216@cts.rutgers.edu (A.T.); jc.sankey@rutgers.edu (J.C.-S.); og96@cts.rutgers.edu (O.G.); delnevo@cts.rutgers.edu (C.D.D.); wackowol@cts.rutgers.edu (O.A.W.); 2 Department of Health Behavior, Society and Policy, Rutgers School of Public Health, Rutgers University, Piscataway, NJ 08854, USA; 3 Perelman School of Medicine, University of Pennsylvania, Philadelphia, PA 19104, USA; strasse3@pennmedicine.upenn.edu

**Keywords:** cigars, warning labels, tobacco, survey methods

## Abstract

Although cigars pose health risks similar to cigarettes, their packaging/marketing is not subject to commensurate regulation in the US. In a 2000 agreement with the Federal Trade Commission, seven major manufacturers agreed to use some form of cigar warning. In 2016, the Food and Drug Administration passed a rule requiring larger standardized warnings, but the requirement was successfully challenged in court. Here, we examined U.S. population-level trends in noticing existing cigarillo, traditional and filtered cigar warnings. We analyzed Wave 5 Population Assessment of Tobacco and Health adult data to assess prevalence of past-30 day warning noticing and associations with socio-demographic and tobacco use variables. Noticing was higher among current users of cigarillos (27%), filtered (34%) and traditional cigars (21%), than non-users (8% for each product, *p* < 0.0001), and among every-day vs. some-day users, established vs. experimental users, and past-30 day users vs. those without past-30 day use. Results varied by product, but generally indicated lower noticing among non-Hispanic Whites and dual cigarette users, but higher noticing among those purchasing cigars by the box/pack (vs. not purchasing for themselves). Low overall noticing but higher prevalence among frequent users underscores a need for a stronger, uniform cigar warning label policy in the US.

## 1. Introduction

As combustible tobacco products, cigars pose similar health risks to those of cigarettes, including respiratory and cardiovascular disease as well as high levels of exposure to carcinogenic compounds [1,2]. In the United States (U.S.), cigar products are available in three primary types—large cigars, cigarillos, and filtered (little) cigars—and vary in features including size, weight, flavors, and quantity sold per package [3]. Of special concern is that cigar smoking is particularly prevalent in potentially vulnerable populations in the U.S. including young adults and minority groups (e.g., non-Hispanic Black and Hispanic) [4,5], with young adults representing the majority of cigarillo smokers [6]. Individuals with lower education levels and those living below the poverty level also account for a significant portion of cigar use [6].

Given the health risks of tobacco use, tobacco warning labels are one tool used by policy makers and regulators to inform consumers and discourage product use. Notably, cigarette packaging has been required to carry a warning label in the U.S. since the 1965 passage of the Federal Cigarette Labeling and Advertising Act, and, per requirements of the 2009 Family Smoking Prevention and Tobacco Control Act (TCA), the U.S. Food and Drug Administration (FDA) published a new rule in 2020 to adopt larger graphic warning labels on cigarette packaging [7,8]. Yet cigar packaging is not subject to a commensurate universal warning requirement. In 2000, pursuant to an agreement with the Federal Trade Commission (FTC), the seven largest cigar manufacturers in the U.S. agreed to display one warning from a set of approved warning statements on cigar advertising and packaging [9]. When the FDA passed a “deeming rule” in 2016 that extended its tobacco regulatory authority to additional products including cigars, it further required health warnings to be placed on all cigar packaging, and for these to comprise 30 percent of both primary display panels (i.e., front and back of pack), representing an increase in size and prominence from the prior labels [10,11,12]. However, representatives of the cigar industry challenged the new requirement, and the US Court of Appeals for the D.C. Circuit [13] found that the warning requirement did not meet the standards of the TCA because the FDA had not sufficiently established potential impacts of the rule on smoking cessation or reduced initiation, as is required by the Act [14]. As such, the FDA’s deeming rule cigar warning requirements are not currently enforced, although the FDA has indicated cigar companies can follow the guidelines voluntarily.

Following the legal challenge, utilization of warning labels on cigar packaging has been inconsistent. One study of warning label compliance conducted in California found that warning compliance varied considerably. Approximately one-third of cigar packs had warnings that did not meet Deeming Rule standards (i.e., warnings that did not comprise 30% of both primary display panels), or no warnings at all. Compliance differed significantly by brand, with brands such as Swisher Sweets reaching over 90% compliance vs. 20% for brands including Dutch Masters. Compliance also varied by product type, with lowest compliance found on cigarillos (over 1/3 had a warning of less than 30% and only on one panel) and on packs of three, with packs less than three also having lower compliance than larger packs of 20 (the latter of which had the highest compliance, all displaying 30% labels on both primary display panels) [15]. Despite voluntary compliance with the deeming rule standards by some cigar manufacturers, cigar labeling in the US lacks a universal, consistent warning requirement.

Importantly, cigarette warnings have been shown to be effective tools in combating cigarette smoking and helping to depress smoking rates [16,17]. Youth and adult smokers report that tobacco warning labels have turned them off from smoking, inspired them to smoke less or prompted cessation attempts, and non-smokers state that warning labels have discouraged them from smoking [18,19,20,21]. Research has also associated processing warnings with subsequent increases in quit intentions and cessation attempts [22,23], and smokers and non-smokers credit cigarette warnings as a source of risk information [16].

Still, the effectiveness of warnings is contingent on processing the warning. Attention to any persuasive message is necessary for that message’s efficacy [24,25]. For warning labels, noticing is a prerequisite to effects [26]: “before a warning label can help a consumer better understand and appreciate the risks against which it warns, the consumer must notice and pay attention to the warning” [12] (p. 28989). Prior reviews of warning effectiveness have found that strengthening warnings (e.g., via changes in size, graphics, content) leads to increased attention to the warnings [17,26].

While there is a substantial body of research concerning cigarette warning labels, research pertaining to cigar warning labels is more limited. Studies thus far have indicated utility of different strategies for potentially improving cigar warnings, such as incorporating pictorials [27,28,29] or emphasizing cardiovascular and respiratory health effects [30,31,32]. Other research indicates that pack factors such as background color and personal characteristics such as susceptibility to smoking and prior product use can influence warning awareness and response [33,34]. However, there is a lack of basic data on cigar warning label exposure/noticing for the three distinct types of cigar products (which have different user profiles and packaging characteristics) [6], evincing a need for additional research. Considering the potential for warnings to promote desired tobacco control outcomes, the importance of warning noticing/attention, and the current lack of a standard warning requirement for cigar packaging and advertising in the U.S., we sought to examine population-level trends in noticing existing cigar warning labels for each of the three major types of cigar products. Using data from the Population Assessment of Tobacco and Health (PATH) study, the aim of the study was to assess the prevalence of noticing warning labels on different types of cigar products in the U.S., as well as potential associations between noticing and tobacco use patterns, purchase behaviors, and/or user demographics; the study documented significant associations between smokers’ reported noticing and their demographic/use characteristics.

## 2. Materials and Methods

### 2.1. Study Sample

This study used data from the Wave 5 adult survey public-use file of the PATH Study, which includes nationally representative, longitudinal cohorts of civilian, non-institutionalized youth and adults in the US [35]. The PATH Study collects information on tobacco use and health status through audio computer-assisted self-interviews in English and Spanish [35]. The Wave 5 survey of the PATH Study was collected between December 2018 and November 2019. The weighted response rates of the PATH study’s adult survey was 74.0% at Wave 1 and 69.4% at Wave 5, among Wave 1 respondents. More details about the PATH Study can be found elsewhere [35,36,37]. For this analysis, we used the sample of the entire US adult population (age ≥ 18 years).

### 2.2. Measures

#### 2.2.1. Cigar Product Use

Current users of each cigar product type were identified using the derived variable for “Adult current every day/some day” (EDSD) smokers of each cigar type. Respondents who identified as an EDSD user indicated that they had ever smoked the respective product and that they currently smoke it every day or some days. Respondents coded as a “no” response on this variable were identified as non-users. This variable does not include users who only smoke cigarillos as blunts, but can include users who only smoke any other type of cigars as blunts. For use frequency, respondents who indicated smoking every day were considered “every-day users”, and those who indicated smoking some days were considered “some-day users” (based on the question “Do you now smoke [cigar product]?”). EDSD users of each product type who indicated they had ever smoked the respective product fairly regularly were considered “established” users, whereas those who indicated they had never smoked the product fairly regularly were considered “experimental” users (based on the derived variable “adult [cigar product] lifetime threshold of use”). Past-30 day product use was identified using the variable “Wave 5 adult past 30 day [cigar product] smoker”, which referred to respondents who indicated they had smoked the product type within the past 30 days.

#### 2.2.2. Noticing Cigar Warnings

Noticing cigar warnings was assessed by the question (asked to all adult respondents), “In the past 30 days, how often, if at all, have you noticed the health warnings on packages of [Cigar Product]?”, for each cigar type. For this analysis, respondents who indicated “Sometimes”, “Often”, or “Very often” were considered to have noticed the warnings, while those who chose “Never” or “Rarely” were considered not to have noticed the warnings.

#### 2.2.3. Other Tobacco Use Patterns

Current use of cigarettes was defined by the derived variable for “Wave 5 adult current every day/some day cigarette smoker”, which included respondents who indicated they had ever smoked a cigarette and now smoke cigarettes every day or some days. Similarly, current use of e-cigarettes was defined by the derived variable for “Wave 5 adult current every day/some day electronic nicotine product smoker”, referring to those who had ever smoked such a product and now smoke every day or some days. Past-12-month blunt smoking was indicated by a response of “Yes” to the question, “In the past 12 months, have you smoked part or all of a traditional cigar, cigarillo, or filtered cigar with marijuana in it?”.

#### 2.2.4. Cigar Purchasing Behavior

Cigar purchasing behavior was measured by two questions for each product type. The first asked, “How do you usually buy [Cigar Product] for yourself?” Respondents who selected “I do not buy my own [Cigar Product]” were considered to not purchase cigars for themselves. Those who indicated purchasing their own cigars (“in person”, “from the internet” or “by telephone”) were also asked, “Do you usually buy [Cigar Product] by the box or pack, or as single [Cigar Product]?”. For analysis, respondents were categorized, based on these two questions, as purchasing by the box/pack, purchasing as singles or not purchasing for themselves.

#### 2.2.5. Covariates

Socio-demographic variables included in the analyses were race/ethnicity (non-Hispanic Black, non-Hispanic White, Hispanic, and non-Hispanic Other), sex (female, male), age (18–34 years, 35–54 years, and ≥55 years), annual household income (<US $25,000, US $25,000–$49,999, US $50,000–$99,999, and ≥US $100,000), and highest educational attainment (<High school/GED, High school graduate, Some college/Associates degree, and Bachelor’s or advanced degree).

### 2.3. Statistical Analysis

We conducted the following statistical analyses using Stata 16.0 [38] in 2021. We used Adult—Wave 5 Cohort Single-Wave Weights with 95% confidence intervals, utilizing the balanced repeated replications (BRR) method with Fay’s adjustment of 0.3 [39]. We first examined the prevalence of noticing cigar warnings among current users versus non-users of each cigar product type. Then, we conducted more focused bivariate analysis of warning noticing among current EDSD users of each cigar product type (i.e., those with more opportunity to be exposed), using Chi-square tests to examine whether noticing varied by socio-demographic variables and additional tobacco use variables; for each variable analyzed, we deleted missing/inapplicable cases on that variable. Lastly, we ran multivariable logistic regressions to assess the associations between noticing cigar warnings and the studied variables. All analyses were conducted individually by cigar product type, deleting missing/inapplicable cases on any variable. When available, we used imputed or derived socio-demographic background measures and current tobacco use measures. For the regression models, we excluded observations with missing values by listwise deletion for multivariable regression [38]. This research only involved the use of de-identified data, which is not considered human subjects research defined under the Department of Health and Human Services regulations 45 CFR 46.102(d).

## 3. Results

### 3.1. Warning Noticing Prevalence

Table 1 presents results regarding past-30 day noticing of warning labels by product type and user type. For each product type, noticing warnings was significantly associated with current use status. The prevalence of noticing warnings was higher among current users of cigarillos (27%), filtered cigars (34%) and traditional cigars (21%), relative to non-users (8% for each product, all *p* < 0.0001).

### 3.2. Correlates of Warning Noticing

Supplementary Table S1 summarizes demographic and usage characteristics of current users, by cigar product type. Table 2 presents bivariate associations of past-30 day warning noticing with demographic and usage variables, among EDSD users of each cigar product type. Key results are described in Section 3.2.1 and Section 3.2.2; prevalence percentages discussed represent significant differences based on chi-square tests for prevalence estimates.

Table 3 presents results of logistic regression analyses for noticing by product type, among EDSD users. Table 3 displays variables with patterns of significant results, but Supplementary Table S2 reports full results for all variables included in the models. Key results are described in Section 3.2.1 and Section 3.2.2; adjusted odds ratios represent significant results of the multivariable logistic regressions.

#### 3.2.1. Demographic Correlates of Warning Noticing

Cigarillos: Noticing cigarillo warnings was significantly associated with race/ethnicity, with higher prevalence of noticing among respondents identifying as non-Hispanic Black (37%) or non-Hispanic Other (33%), compared to those identifying as non-Hispanic White (20%, *p* < 0.001). The odds of noticing among non-Hispanic Black and non-Hispanic Other respondents were approximately twice the odds (respectively 1.94 and 2.06) of Non-Hispanic White respondents.

Filtered cigars: There was no significant association between noticing filtered cigar warnings and any of the studied demographic variables.

Traditional cigars: Noticing traditional cigar warnings was significantly associated with sex and race/ethnicity. A greater proportion of males reported higher noticing than females (respectively, 22% vs. 12%, *p* < 0.01). A greater proportion of non-Hispanic Other respondents reported noticing compared with Hispanic or non-Hispanic White respondents (respectively, 40%; 18%, 19%; *p* < 0.05), with the former having roughly 3.5 times higher odds of noticing than the latter.

#### 3.2.2. Associations of Warning Noticing and Tobacco Usage Patterns

Cigarillos: Noticing prevalence was higher among every-day (46%) versus some-day users (24%), among established (33%) versus experimental users (20%), and among past-30 day users (30%) versus those with no past-30 day use (13%) (all *p* < 0.0001). Every-day users had 2.27 times higher odds of noticing than did some-day users. Cigarillo users who are also cigarette smokers reflected lower prevalence of noticing cigarillo warnings (23%) than cigarette non-smokers (33%, *p* < 0.01), and had 37% lower odds of noticing than non-smokers. Cigarillo users who reported any past-12 month cigar-as-blunt use were more likely to notice cigarillo warnings (33%, versus 22% for those who did not, all *p* < 0.001). Any past-12 month blunt use increased odds of noticing by 57% relative to no use as blunts. Noticing prevalence was higher among respondents who purchased cigarillos by the pack (32%) or as singles (26%) than those who did not buy their own cigarillos (16%, *p* < 0.01); not purchasing their own cigarillos was associated with 60% lower odds of noticing relative to purchasing by the pack.

Filtered cigars: Similar to cigarillos, noticing prevalence was higher among every-day (45%) vs. some-day users (29%, *p* < 0.01), among established (39%) vs. experimental users (27%, *p* < 0.05), and among past-30 day users (37%) vs. those with no past-30 day use (17%, *p* < 0.05). Every-day users had 94% higher odds of noticing than did some-day users. Filtered cigar smokers who also smoke cigarettes had 47% lower odds of noticing filtered cigar warnings than cigarette non-smokers. Respondents who purchased filtered cigars by the pack (37%) or as singles (36%) were more likely to notice warnings than those who did not buy their own cigars (20%, *p* < 0.05).

Traditional cigars: Noticing prevalence was higher among every-day (48%) vs. some-day users (20%, *p* < 0.001), among established (29%) vs. experimental (15%, *p* < 0.0001), and among past-30 day users (26%) vs. those with no past-30 day use, (8%, *p* < 0.0001). Every-day traditional cigar smokers had nearly three times higher odds of noticing than some-day smokers. Further, traditional cigar smokers who also smoke cigarettes had 51% lower odds of noticing than those who do not also smoke cigarettes. Noticing prevalence was higher among respondents who purchased traditional cigars by the box/pack (36%) than those who purchased as singles (20%) or did not buy their own cigars (9%, *p* < 0.0001), and purchasing as singles or not buying their own cigars was associated with 51% and 78% lower odds of noticing, respectively, relative to purchasing by the box/pack.

## 4. Discussion

Our research revealed low overall prevalence of noticing cigar warning labels among users of each cigar type, and considerably lower prevalence among non-users. The lack of a uniform cigar warning requirement and label standards in the US likely contribute to inconsistent application of warning labels and thereby inconsistent opportunity to notice such warnings. Previous research has shown that inclusion of any warning label on cigar packaging or advertising, and compliance with the requirements initially set forth by the deeming rule, differ by brand and individual product, with considerable variation in the use and type of warning labels observed [40,41]. As a result, maximizing cigar users’ awareness of product risks will likely rely on the establishment of a universal warning label standard for cigar packaging in the US.

Yet even with the low overall prevalence, noticing was significantly more common among frequent, recent users across all cigar types. This disparity indicates that there are non-trivial levels of noticing current labels among those users who most need to be aware of product risks as a result of their use. Further, greater noticing among cigarillo and traditional cigar users who purchase by the pack relative to not purchasing for themselves, and traditional cigar users who purchase by the pack rather than as singles, also illustrates this trend (and appears consistent with prior research findings that larger packs are more often compliant with recommended Deeming Rule warning label standards than are singles) [15]. Still, these respondents use cigar products more frequently despite greater warning cognizance (perhaps related to potentially higher exposure to pro-cigar marketing as well, since warnings appear on advertisements and packages), indicating an enduring need for research into cigar warning effectiveness and evolving warning strategies. As warnings on packs/boxes seem to command higher levels of noticing, our findings also suggest the continued relevance of research into regulatory options related to pack size/minimum quantity sold. Products sold as singles may be separated from a warning label carried on the box packaging they are originally found in, and generally provide less surface area for clear warning label display. Furthermore, while we did not compare findings across product type, our descriptive findings support potential variations in warning noticing by product, and in the relative importance of purchasing by the pack or as singles for different products. Future research could benefit from explicitly comparing warning label compliance by product type and packaging/purchase type (e.g., packs vs. singles) and assessing corresponding differences in noticing warnings, compared across different products.

Our findings further indicate that Non-Hispanic Black and/or Non-Hispanic Other respondents are more likely to notice cigarillo and traditional cigar warnings, respectively, than Non-Hispanic White respondents, which may be related to differences in use of these products (research shows that past-30 day cigarillo smoking prevalence is nearly four times higher for Non-Hispanic Black than Non-Hispanic White individuals) [4], as well as other potential factors such as differences in exposure to pro-tobacco marketing (which also often simultaneously displays a warning). Additional research is needed to examine the effects of warning noticing and attention on cigar harm perceptions and ongoing product use intentions, in general and among vulnerable groups in particular given health disparities associated with cigarillo smoking.

Despite evidence to the contrary, cigars are sometimes perceived as less harmful than cigarettes, and misunderstandings persist around the harms of cigar smoking [42,43,44]. In fact, in studies of cigar labeling, participants have found warnings stating that cigars are not a safe alternative to cigarettes to be less believable than other warnings [31,32]. Of concern, our findings indicate that dual cigar/cigarette users are less likely to notice cigar warnings; while this could result from such users having become accustomed or even desensitized to tobacco warnings in general, our data do not allow us to confirm the reason for this finding. Importantly, as evidence shows that the harm of cigar use is commensurate with that of cigarette use (and in some cases, the exposure to carcinogenic compounds may be even higher) [2], so too should be the warning label requirements. In fact, some research indicates that pictorial warnings like those that have been shown to be effective for cigarettes, including graphic ones and ones depicting people, can help to garner attention, promote cognitive processing, and spark emotional reactions when used on cigar packaging [27,28]. Future studies should build upon this emergent research pertaining to promising strategies for cigar labeling. More broadly, given the prevalence of dual/poly tobacco use among consumers, policies should consider warning label strategies for tobacco products widely, as absent or weaker labels on some products (e.g., cigars) versus others (e.g., cigarettes) could unintentionally be perceived as suggestive of differences in product risks.

Our study has some limitations that should be noted. While the PATH study is a longitudinal dataset, our analysis was cross-sectional, with the corresponding weights applied. Further, some analyses had relatively small sample sizes, as they were limited to current users. Another limitation is that our analysis relied on self-report data for warning noticing, which could be subject to faulty recall or to over-reporting resulting from the question phrasing. Future research could pursue experimental methods of assessing warning recognition/recall.

Especially given the disproportionate burden of cigar use that is borne by vulnerable populations [4]—and that availability and marketing of various cigar types are especially common in low-income and minority populations [45,46]—it remains crucial to foster clear communication of product risk. This should include considering how to reach users who do not buy cigars for themselves but are still smoking them, potentially in social settings that can promote continued or escalated use; such efforts may entail consideration of larger pictorial warnings, restrictions of smaller pack sizes, and public health education/campaigns. Strengthening policy for cigar warning requirements can promote warning noticing and, in turn, other targeted outcomes; the effects of enhanced warning labels on cigarette warning attention have been shown to trickle down to effects on quit attempts and decreased smoking prevalence [17,26]. Our research illustrates a need and an opportunity for tobacco control policy in the U.S. to implement standard warning requirements to promote awareness of cigar risks.

## 5. Conclusions

Understanding trends related to warning noticing helps to underscore a role of uniform cigar warning policy in the U.S. and provides important baseline data on this topic. The current research illustrates insufficient rates of cigar warning label noticing among users and highlights distinct patterns in warning noticing by demographics, usage characteristics, and purchase behaviors. Ultimately, our findings point to an overall need for improved warning noticing and demonstrate that current noticing is higher among regular users and among purchasers of packs, which may indicate that restrictions on product packaging (e.g., minimum quantities sold) and implementation of standard, prominent warnings for all cigar products may enhance warning noticing and support communication of product risks.

## Figures and Tables

**Table 1:** Weighted Prevalence of past 30-day warning noticing among every-day/some-day cigar product users and non-users, by product type.

	Cigarillo		Filtered Cigar		Traditional Cigar	
	User Noticing	Non-user Noticing	User Noticing	Non-user Noticing	User Noticing	Non-user Noticing
	(%, CI)	(%, CI)	(%, CI)	(%, CI)	(%, CI)	(%, CI)
	(n = 1385)	(n = 31,160)	(n = 675)	(n = 31,865)	(n = 1361)	(n = 31,179)
Never	51.96%	83.76%	45.76%	84.22%	61.77%	83.51%
	(48.41, 55.49)	(83.06, 84.44)	(40.84, 50.77)	(83.57, 84.84)	(57.81, 65.58)	(82.81, 84.19)
Rarely	20.85%	7.88%	20.51%	7.63%	17.2%	8.81%
	(18.24, 23.74)	(7.42, 8.37)	(17.24, 24.21)	(7.16, 8.13)	(14.94, 19.72)	(8.33, 9.32)
Sometimes	14.24%	3.97%	15.41%	3.89%	12.88%	3.8%
	(12.12, 16.67)	(3.66, 4.3)	(1.25, 18.85)	(3.58, 4.22)	(10.56, 15.61)	(3.48, 4.15)
Often	7.47%	2.2%	9.93%	2.08%	5.62%	1.91%
	(5.93, 9.37)	(1.97, 2.46)	(7.39, 13.22)	(1.87, 2.31)	(3.96, 7.9)	(1.67, 2.19)
Very often	5.48%	2.19%	8.39%	2.18%	2.54%	1.96%
	(4.01, 7.44)	(1.98, 2.43)	(5.97, 11.67)	(1.94, 2.45)	(1.69, 3.8)	(1.77, 2.17)
At least sometimes ^1^	27.19%	8.36%	33.73%	8.15%	21.03%	7.67%
	(24.07, 30.54)	(7.9, 8.82)	(29.83, 37.85)	(7.67, 8.65)	(17.82, 24.64)	(7.22, 8.15)

^1^ This includes respondents answering sometimes, often, or very often.

**Table 2:** Weighted Prevalence of past 30-day warning noticing among every day/some day cigar product users and non-users, by demographics and tobacco use.

	Cigarillo	Filtered Cigar	Traditional Cigar
	% Noticing (CI)	% Noticing (CI)	% Noticing (CI)
**Sex**	(n = 1383) (ns)	(n = 673) (ns)	(n = 1359) (*p* < 0.01)
male	25.75% (22.35, 29.48)	33.34% (28.42, 38.65)	22.35% (18.70, 26.49)
female	30.73% (24.92, 37.21)	34.67% (26.95, 43.29)	12.37% (08.31, 18.03)
**Age**	(n = 1385) (ns)	(n = 675) (ns)	(n = 1361) (ns)
18–34	30.97% (26.46, 35.87)	28.51% (21.66, 36.52)	20.95% (17.16, 25.33)
35–54	23.42% (18.66, 28.98)	32.92% (25.61, 41.16)	20.67% (15.33, 27.27)
55+	23.12% (16.09, 32.06)	43.53% (34.97, 52.49)	21.64% (15.47, 29.41)
**Race/Ethnicity**	(n = 1372) (*p* < 0.001)	(n = 663) (ns)	(n = 1342) (*p* < 0.05)
Non-Hispanic (NH) White	19.55% (15.7, 24.09)	30.46% (25.09, 36.41)	18.86% (14.93, 23.53)
NH Black	36.5% (30.55, 42.89)	36.85% (28.69, 45.84)	24.96% (18.37, 32.96)
NH Other	32.74% (22.01, 45.64)	21.13% (10.24, 38.60)	39.91% (24.67, 57.39)
Hispanic	28.21% (20.06, 38.1)	36.18% (23.64, 50.94)	17.55% (10.41, 28.04)
	Significant: NH White vs. NH Black and NH Other		Significant: NH Other vs. NH White and Hispanic
**Education**	(n = 1379) (ns)	(n = 673) (ns)	(n = 1355) (*p* < 0.05)
Less than high school/GED	29.12% (23.03, 36.07)	41.55% (34.06, 49.45)	22.47% (16.08, 30.48)
High school graduate	23% (18.48, 28.24)	28.67% (21.13, 37.63)	28.96% (21.13, 38.30)
Some college (no degree) or Associates	30.66% (25.41, 36.47)	33.6% (26.03, 42.11)	15.86% (12.44, 19.99)
Bachelor’s degree or advanced degree	23.84% (14.92, 35.84)	25.69% (12.44, 45.68)	20.97% (14.94, 28.60)
			Significant: HS vs. Some College
**Income**	(n = 1321) (ns)	(n = 636) (ns)	(n = 1317) (ns)
less than $24,999	30.22% (26.24, 34.52)	37.8% (32.34, 43.58)	28.15% (21.98, 35.27)
25,000–49,999	25.86% (19.36, 33.64)	21.62% (14.18, 31.54)	16.47% (10.86, 24.18)
50,000–99,999	27.46% (20.26, 36.05)	38.05% (22.60, 56.37)	22.75% (17.44, 29.11)
100,000 or more	20.48% (11.88, 32.97)	23.15% (10.06, 44.79)	19.52% (13.01, 28.23)
**Type of user**	(n = 1384) (*p* < 0.0001)	(n = 674) (*p* < 0.05)	(n = 1361) (*p* < 0.0001)
established	32.26% (28.14, 36.67)	38.38% (33.72, 43.26)	28.79% (23.71, 34.47)
experimental	19.76% (16.25, 23.81)	26.86% (20.15, 34.82)	15.2% (11.97, 19.11)
**Use frequency**	(n = 1385) (*p* < 0.0001)	(n = 675) (*p* < 0.01)	(n = 1361) (*p* < 0.001)
every day	45.66% (36.60, 55.02)	45.39% (36.68, 54.40)	48.25% (30.02, 66.95)
some days	23.98% (21.05, 27.17)	29.09% (24.22, 34.49)	19.53% (16.58, 22.87)
**Past-30 day product use**	(n = 1385) (*p* < 0.0001)	(n = 675) (*p* < 0.05)	(n = 1361) (*p* < 0.0001)
yes	29.57% (25.92, 33.51)	36.85% (32.78, 41.12)	26.45% (22.23, 31.15)
no	13.37% (09.42, 18.65)	17.18% (08.60, 31.38)	8.1% (05.80, 11.22)
**Current cigarette smoker**	(n = 1382) (*p* < 0.01)	(n = 674) (ns)	(n = 1359) (ns)
yes	23.06% (19.54, 27)	32.2% (28.23, 36.43)	18.76% (14.08, 24.54)
no	33.23% (27.83, 39.11)	39.5% (27.96, 52.33)	22.26% (17.73, 27.55)
**Current e-cigarette user**	(n = 1385) (ns)	(n = 675) (ns)	(n = 1361) (ns)
yes	24.48% (20.38, 29.09)	29.71% (22.97, 37.47)	26.55% (19.79, 34.61)
no	28.28% (24.48, 32.42)	35.46% (30.11, 41.19)	19.71% (16.00, 24.02)
**Past-12 month blunt use**	(n = 1381) (*p* < 0.001)	(n = 673) (ns)	(n = 1359) (ns)
yes	33.2% (28.72, 38.01)	32.85% (27.12, 39.14)	24.23% (18.23, 31.44)
no	21.71% (17.76, 26.26)	34.39% (28.16, 41.20)	19.88% (16.30, 24.02)
**Cigar purchase type**	(n = 1378) (*p* < 0.01)	(n = 667) (*p* < 0.05)	(n = 1355) (*p* < 0.0001)
Box	31.52% (26.4, 37.1)	36.79% (31.41, 42.53)	35.62% (28.35, 43.61)
Single	26.45% (22.17, 31.21)	35.54% (2.79, 44.02)	19.63% (15.83, 24.07)
Don’t by own cigars	15.59% (09.83, 23.84)	19.68% (10.63, 33.55)	9.23% (04.56, 17.79)
	Significant: Don’t by own cigars vs. Pack and Single	Significant: Don’t by own cigars vs. Pack and Single	Significant: All categories vs. all else

Note: ns = not significant at *p* < 0.05; all significant *p*-values indicated.

**Table 3:** Weighted Logistic regression: Noticing cigar warnings by demographics/use, among every-day/some-day product users.

	Cigarillo Warning Noticing (n = 1294)		Filtered Cigar Warning Noticing (n = 615)		Traditional Cigar Warning Noticing (n = 1292)	
Variable	Odds Ratio (CI)	*p*-value	Odds Ratio	*p*-value	Odds Ratio	*p*-value
**Race/Ethnicity**						
NH White	ref	ref	ref	ref	ref	ref
NH Black	**1.94 (1.30, 2.89)**	**0.001**	1.38 (0.81, 2.37)	0.23	1.21 (0.68, 2.17)	0.51
Hispanic	1.70 (0.99, 2.93)	0.053	1.68 (0.83, 3.39)	0.14	1.04 (0.47, 2.26)	0.93
NH Other	**2.06 (1.03, 4.13)**	**0.042**	0.69 (0.23, 2.02)	0.49	**3.52 (1.61, 7.72)**	**0.002**
**Use Frequency**						
some days	ref	ref	ref	ref	ref	ref
every day	**2.27 (1.40, 3.69)**	**0.001**	**1.94 (1.13, 3.33)**	**0.02**	**2.95 (1.02, 8.51)**	**0.045**
**Current Cigarette Smoker**						
no	ref	ref	ref	ref	ref	ref
yes	**0.63 (0.43, 0.92)**	**0.017**	**0.53 (0.29, 0.98)**	**0.04**	**0.49 (0.29, 0.83)**	**0.009**
**Past-12 Month Blunt Use**						
no	ref	ref	ref	ref	ref	ref
yes	**1.57 (1.10, 2.25)**	**0.014**	1.13 (0.62, 2.08)	0.68	1.17 (0.69, 2.00)	0.55
**Cigar Purchase Type**						
Box/pack (in person)	ref	ref	ref	ref	ref	ref
Not in person	**0.40 (0.21, 0.77)**	**0.007**	0.45 (0.19, 1.04)	0.06	**0.22 (0.08, 0.57)**	**0.002**
Singles (in person)	0.71 (0.50, 1.01)	0.058	0.98 (0.57, 1.66)	0.93	**0.49 (0.32, 0.76)**	**0.002**

Note: Values in bold indicate significant difference from the reference category. These models also included the variables of sex, age, education, income, and current e-cigarette use, which, overall, were not significantly associated with warning noticing. For full results, see Supplementary Table S2.

## Data Availability

The coding materials for this article can be made available upon reasonable request.

## Supplementary Materials

The following are available online at https://www.mdpi.com/article/10.3390/ijerph19063221/s1, Table S1: Demographic and usage characteristics of current every-day/some-day users, by product type; Table S2: Weighted Logistic regression: Noticing cigar warnings by demographics/use, among every-day/some-day product users.
