# Supplementary material for: Cigar Warning Noticing and Demographic and Usage Correlates: Analysis from the United States Population Assessment of Tobacco and Health Study, Wave 5
Source: Int J Environ Res Public Health. 2022 Mar 9;19(6):3221. doi: 10.3390/ijerph19063221 (PMC8952384; doi:10.3390/ijerph19063221)
Supplement: Supplementary file 1 [file ijerph-19-03221-s001.zip › ijerph-1599504-supplementary.pdf]

International Journal of
Environmental Research and Public Health

MDPI

Supplementary Materials

Table S1. Demographic and usage characteristics of current every-day/some-day users, by product type.

	Cigarillo	Filtered Cigar	Traditional Cigar
**Sex**	(n=1383)	(n=673)	(n=1359)
male	70.65%	66.72%	86.97%
**Age**	(n=1385)	(n=675)	(n=1361)
18-34	50.50%	40.93%	36.31%
35-54	33.92%	34.48%	37.26%
55+	15.57%	24.60%	26.43%
**Race/Ethnicity**	(n=1372)	(n=663)	(n=1342)
Non-Hispanic (NH) White	47.78%	50.23%	66.40%
NH Black	31.38%	23.38%	13.43%
NH Other	5.48%	6.49%	6.88%
Hispanic	15.37%	19.90%	13.29%
**Education**	(n=1379)	(n=673)	(n=1355)
Less than high school/GED	23.46%	30.69%	12.53%
High school graduate	27.93%	32.80%	20.12%
Some college (no degree) or Associates	35.36%	26.12%	33.11%
Bachelor's degree or advanced degree	13.25%	10.40%	34.24%
**Income**	(n=1321)	(n=636)	(n=1317)
less than $24,999	46.95%	62.36%	21.65%
25,000-49,999	23.32%	21.85%	21.01%
50,000-99,999	18.75%	11.41%	24.97%
100,000 or more	10.98%	4.37%	32.37%
**Type of user**	(n=1384)	(n=674)	(n=1361)
Established	59.46%	59.90%	42.89%
Experimental	40.54%	40.10%	57.11%
**Use frequency**	(n=1385)	(n=675)	(n=1361)
every day	14.81%	28.43%	5.20%
some days	85.19%	71.57%	94.80%
**Past-30 day product use**	(n=1385)	(n=675)	(n=1361)
yes	85.28%	84.11%	70.45%
**Current cigarette smoker**	(n=1382)	(n=674)	(n=1359)
yes	59.54%	78.58%	35.72%
**Current e-cig user**	(n=1385)	(n=675)	(n=1361)
yes	28.81%	30.08%	19.33%
**Past 12-month any cigar use as blunts**	(n=1381)	(n=673)	(n=1359)
yes	47.71%	43.21%	26.85%
**Cigar purchase type**	(n=1378)	(n=667)	(n=1355)
Box	42.69%	59.51%	21.66%
Single	46.02%	25.78%	59.22%
Don't by own cigars	11.29%	14.70%	19.12%

Table S2. Weighted Logistic regression: Noticing cigar warnings by demographics/use, among every-day/some-day product users.

	**Cigarillo Warning Noticing (n = 1294)**		**Filtered Cigar Warning Noticing (n = 615)**		**Traditional Cigar Warning Noticing (n = 1292)**	
Variable	Odds Ratio (CI)	*p*- value	Odds Ratio	*p*- value	Odds Ratio	*p*- value
**Sex**						
male	ref	ref	ref	ref	ref	ref
female	1.05 (0.74, 1.5)	0.80	1.33 (.76, 2.30)	0.31	.57 (.31, 1.06)	0.08
**Age**						
18-34	ref	ref	ref	ref	ref	ref
35-54	**0.64 (0.43, 0.93)**	**0.021**	1.13 (.64, 1.98)	0.67	.99 (.62, 1.59)	0.97
55+	0.62 (0.33, 1.17)	0.14	1.55 (.78, 3.08)	0.21	.84 (.50, 1.42)	0.52
**Race/Ethnicity**						
NH White	ref	ref	ref	ref	ref	ref
NH Black	**1.94 (1.30, 2.89)**	**0.001**	1.38 (.81, 2.37)	0.23	1.21 (.68, 2.17)	0.51
Hispanic	1.70 (.99, 2.93)	0.053	1.68 (.83, 3.39)	0.14	1.04 (.47, 2.26)	0.93
NH Other	**2.06 (1.03, 4.13)**	**0.042**	.69 (.23, 2.02)	0.49	**3.52 (1.61, 7.72)**	**0.002**
**Education**						
Less than high school/GED	ref	ref	ref	ref	ref	ref
High school graduate	0.76 (.49, 1.18)	0.22	0.79 (.44, 1.43)	0.43	1.61 (.87, 2.97)	0.13
Some college/Associates	1.09 (.69, 1.71)	0.72	.88 (.45, 1.75)	0.72	.77 (.43, 1.38)	0.37
Bachelor's/advanced degree	0.79 (.38, 1.67)	0.54	.57 (.20, 1.64)	0.30	1.12 (.59, 2.10)	0.73
**Income**						
less than $24,999	ref	ref	ref	ref	ref	ref
25,000-49,999	0.73 (.47, 1.12)	0.14	**.47 (.25, .90)**	**0.02**	.58 (.34, 1.00)	0.051
50,000-99,999	1.08 (.64, 1.85)	0.76	1.23 (.48, 3.11)	0.66	.82 (.45, 1.50)	0.51
100,000 or more	0.72 (.35, 1.47)	0.36	.67 (.16, 2.71)	0.57	.56 (.30, 1.05)	0.07
**Use Frequency**						
some days	ref	ref	ref	ref	ref	ref
every day	**2.27 (1.40, 3.69)**	**0.001**	**1.94 (1.13, 3.33)**	**0.02**	**2.95 (1.02, 8.51)**	**0.045**
**Current Cigarette Smoker**						
no	ref	ref	ref	ref	ref	ref
yes	**.63 (.43, .92)**	**0.017**	**.53 (.29, .98)**	**0.04**	**.49 (.29, .83)**	**0.009**
**Current E-Cigarette Smoker**						
no	ref	ref	ref	ref	ref	ref
yes	.76 (.56, 1.04)	0.09	1.00 (.60, 1.67)	0.996	1.35 (.83, 2.17)	0.22
**Past-12 Month Blunt Use**						
no	ref	ref	ref	ref	ref	ref
yes	**1.57 (1.10, 2.25)**	**0.014**	1.13 (.62, 2.08)	0.68	1.17 (.69, 2.00)	0.55
**Cigar Purchase Type**						
Box/pack (in person)	ref	ref	ref	ref	ref	ref
Not in person	**0.40 (.21, .77)**	**0.007**	0.45 (.19, 1.04)	0.06	**.22 (.08, .57)**	**0.002**
Singles (in person)	0.71 (.50, 1.01)	0.058	0.98 (.57, 1.66)	0.93	**.49 (.32, .76)**	**0.002**
